# Feasibility of the PalliSupport care pathway: results from a mixed-method study in acutely hospitalized older patients at the end of life

**DOI:** 10.1186/s40814-020-00676-0

**Published:** 2020-09-15

**Authors:** Isabelle Flierman, Marjon van Rijn, Marike de Meij, Marjolein Poels, Dorende M. Niezink, Dick L. Willems, Bianca M. Buurman

**Affiliations:** 1 Amsterdam UMC, Department of General Practice, Section of Medical Ethics, Amsterdam Public Health research institute, University of Amsterdam, Meibergdreef 9, Amsterdam, The Netherlands; 2 Amsterdam UMC, Department of Internal Medicine, Section of Geriatric Medicine, Amsterdam Public Health research institute, University of Amsterdam, Meibergdreef 9, Amsterdam, The Netherlands; 3 Center of Expertise Urban Vitality, Faculty of Health, Amsterdam University of Applied Science, Amsterdam, the Netherlands; 4 OLVG, Palliative and Supportive Care Team, Oncology Centre Amsterdam, Oosterpark 9, Amsterdam, The Netherlands; 5 Care2research, Mattenbiesstraat 133, Amsterdam, the Netherlands

**Keywords:** Palliative care, Feasibility study, Transitional care, Hospital care, Older patients

## Abstract

**Background:**

A transitional care pathway (TCP) could improve care for older patients in the last months of life. However, barriers exist such as unidentified palliative care needs and suboptimal collaboration between care settings. The aim of this study was to determine the feasibility of a TCP, named PalliSupport, for older patients at the end of life, prior to a stepped-wedge randomized controlled trial.

**Methods:**

A mixed-method feasibility study was conducted at one hospital with affiliated primary care. Patients were ≥ 60 years and acutely hospitalized. The intervention consisted of (1) training on early identification of the palliative phase and end of life conversations, (2) involvement of a transitional palliative care team during admission and post-discharge and (3) intensified collaboration between care settings. Outcomes were feasibility of recruitment, data collection, patient burden and protocol adherence. Experiences of 14 professionals were assessed through qualitative interviews.

**Results:**

Only 16% of anticipated participants were included which resulted in difficulty assessing other feasibility criteria. The qualitative analysis identified misunderstandings about palliative care, uncertainty about professionals’ roles and difficulties in initiating end of life conversations as barriers. The training program was well received and professionals found the intensified collaboration beneficial for patient care. The patients that participated experienced low burden and data collection on primary outcomes and protocol adherence seems feasible.

**Discussion:**

This study highlights the importance of performing a feasibility study prior to embarking on effectiveness studies. Moving forward, the PalliSupport care pathway will be adjusted to incorporate a more active recruitment approach, additional training on identification and palliative care, and further improvement on data collection.

## Key messages regarding feasibility

- What uncertainties existed regarding the feasibility?

A novel transitional care pathway was developed consisting of multiple components on different levels. Prior to a stepped-wedge RCT to assess effectiveness of the pathway on reduction of unplanned rehospitalizations, we aimed to assess feasibility of the study protocol on recruitment, data collection, patient burden and protocol adherence. Furthermore, acceptability of the intervention by professionals was assessed.

- What are the key feasibility findings?

The protocol in its current form proved not to be feasible because of the low recruitment rate. Practical barriers were uncovered that hindered protocol adherence, and data collection of secondary outcomes was hampered due to deteriorating health of participants. The training program was well received and professionals found the intensified collaboration beneficial for patient care and patients experienced low burden.

- What are the implications of the feasibility findings for the design of the main study?

The care pathway will be adjusted to incorporate a more active recruitment approach and additional training on identification of patients with palliative care needs. To overcome the difficulties in obtaining questionnaires when patients are in the last days of life, a questionnaire for relatives will be added. Further efforts will be made to enhance collaboration between primary and secondary care. A rigorous mixed-method process evaluation according to the MRC framework will be performed alongside the stepped wedge RCT.

## Background

With the ageing population and growing number of people living with, and dying from, chronic diseases, the need for well-organized palliative care is increasingly urgent [1]. Currently, palliative care for older patients is hindered because of multiple barriers: lack of timely identification of palliative needs [2], infrequent conversation about goals of care [3, 4], insufficient collaboration between care professionals [5, 6] and little caregiver support [7, 8]. These barriers can result in unwanted care transitions, mainly acute hospitalizations, and patients not dying at their preferred place [9–13]. A transitional palliative care intervention could overcome these barriers through early identification of needs, advance care planning, symptom management and coordination of care [14].

For this purpose, the PalliSupport care pathway was developed that aims to provide patient centred, goal-oriented care throughout different care settings for older patients and their relatives in the last year of life. The starting point of the pathway is an acute hospitalization, because up to 35% of older patients die in the following year [15], and hospitalizations can often result from unidentified palliative care needs [15–17]. The PalliSupport care pathway was developed according to the MRC-framework [18]. During phase one, the development stage of the intervention, we performed qualitative studies to assess barriers to timely identification of palliative care needs [19] and barriers leading to transitions between community care and the hospital for patients with palliative care needs [20]. Furthermore, in as of yet unpublished studies, we assessed instruments that could aid care professionals in identifying patients in the last year of life and explored the effectiveness of collaborative palliative care teams. In collaboration with experts and after studying best practices, we developed the training modules and established the steps of the pathway. This led to the PalliSupport transitional care pathway consisting of training modules on early identification, advance care planning, protocols on interprofessional and transitional collaboration and establishment of a transitional palliative care team.

Currently, the effectiveness of the pathway is being studied in an ongoing stepped-wedge randomized controlled trial (RCT). In order to assess the effectiveness of the pathway two outcomes were chosen: (1) hospital admissions and (2) number of patients dying at their preferred place of death. These outcomes can be seen as indicators of good symptom control by reducing the need for unplanned hospitalizations and organization of care in such a manner that patients’ wish to die at their preferred place can be fulfilled. Prior to the stepped-wedge RCT, we performed a mixed-method feasibility study, which we present here [21]. The specific objectives of this study were:

1. To determine feasibility of the study protocol on

- Patient and informal caregiver recruitment
- Data collection
- Patient burden
- Implementation of study components and protocol adherence

2) To assess the experiences of professionals with the training module and the care pathway to determine acceptability of the intervention.

## Methods

### Study design

This was a mixed-method feasibility study set in the Netherlands in one urban hospital and affiliated primary care facilities, such as general practitioners offices, community care organizations and care homes. Patients were recruited between February and July 2018 and followed for 6 months after discharge. During the same period, care professionals who were involved in the intervention were interviewed. For the qualitative data, a qualitative description approach was used [22], for this allowed us to acquire firsthand knowledge of professionals’ experience with the intervention [23]. The CONSORT checklist for extension for randomized pilot and feasibility trials was followed for reporting this study [24]. However, this was not a randomized feasibility study, thus not all criteria applied.

### Participants and recruitment

We aimed to include 50 patients in the pre-set duration of this study (6 months) for this meant one inclusion per week per department. Eligible patients were ≥ 60 years and acutely hospitalized for at least 48 h at the department of pulmonology or gastroenterology. We selected these departments because we aimed to include patients with a variety of diagnoses and not solely cancer. However, due to the low inclusion rates, we decided also to recruit from the oncology department during the last 2 months of the study. Presence of palliative care needs was defined as a positive *Surprise Question*, “Would I be surprised if the patient died in the next 12 months?”, and the presence of two or more *Supportive and Palliative Care Indicators Tool*™ (SPICT™) criteria, which include amongst others decline in functional status, repeated unplanned hospital admissions and significant weight loss in the last 3 to 6 months [25, 26]. Patients who lived outside a set postal code area who were cognitive impaired (Mini-Mental State Examination < 15) or did not speak Dutch were excluded. Furthermore, if patients had an informal caregiver, the caregiver was asked to participate in the study to assess caregiver burden. Only informal caregivers that provided more than 8 h of care per week, were 18 years or older and were able to answer Dutch questionnaires could participate. Participation of an informal caregiver was not a prerequisite for patient participation.

Department-based residents and nurses informed the transitional palliative care team (TPCT) if they identified patients with palliative needs and a TPCT member then approached each eligible patient for participation. There was no active recruitment from the researchers. Due to privacy laws in the participating hospital, we were not allowed to assess data on eligible patients and only on those approached by the palliative care team.

### Interventions

Interventions were done on (A) department level and (B) palliative care team level. Table 1 shows the different steps of the pathway and who performs them. Because the aim of the study was to assess feasibility, no control group was used. In the ongoing RCT, the care pathway is being compared to usual care. Usual care entails generalist care with on demand specialist palliative care services in the hospital without follow-up in home-setting and without intensified collaboration between care settings.

**Table 1:** Components of the intervention

Intervention	Components	Intervention conducted by
Identification of palliative care needs during admission	• Identification of palliative needs based on Surprise Question and ≥ 2 SPICT criteria* • Palliative Care team is consulted	Department nurses and physicians
Palliative care assessment and advance care planning	• Assessment of needs, preferences and symptoms on (1) physical, (2) psychological, (3) social and (4) spiritual level • Discussion of treatment limitations^+^ • Discussion of preferred place of death^+^ • Formulating individualized care plan^+^	Palliative care team and/or department physician
Multidisciplinary team meeting	• Weekly discussions about patients with the palliative core team, hospital specialists and non-medical specialist • Invitation GP and community nurse (either in person or by phone)* • Discussing individualized care plan* • The complexity of the patient’s palliative care situation is assessed using the new working methods (colour coding indicating the stability and severity of the problems) *	Palliative care team, department physician, GP, community nurse
Discharge	• Patient receives individualized care plan* • Informal caregiver receives information sheet about support*	Palliative care team or department physician/nurse
Handover	• Contact with GP at least once prior to discharge/during MDT meeting^+^ • MDT summary is sent to GP and/or community nurse within 24 h of discharge^+^ • Medial handover is send to GP within 24 h of discharge^+^	Palliative care team and/or department physician/nurse
Home visit and follow-up	• Home visits at place of care* If applicable • Follow-up discussion at MDT* • Adjustment of individualized care plan* • Adjustment of colour coding*	Palliative care team

*Components that were completely new within the intervention

^+^Components that were already performed for some patients but should be done for all patients during the study

#### Intervention on department level

We gave presentations about early identification for nurses and physicians at the participating departments. In addition, we hosted a practical and interactive training module, spread out over two afternoons and aimed at both nurses and physicians, on how to initiate end of life conversations (in Dutch: STEM-training). The training incorporated discussions about the participants’ personal values regarding the end of life but also addressed how different types of patients tend to have different preferences when it comes to talking about the end of life.

#### Intervention on palliative care team level

Prior to the study, a palliative care team consisting of two clinical nurse specialists, a specialized general practitioner (GP), an oncologist and GPs in training, worked on a consultation basis within the hospital. Patients for whom they were consulted were mostly patients with cancer and on average they were consulted 17 days prior to death. Team members performed palliative assessments and advance care planning conversations and patients were discussed within multidisciplinary team meetings (MDTs) where other medical specialists and non-medical specialists were present. The team was available for patients during office hours and did not follow-up in the home-setting.

To enable the team to work transitionally, changes were made to the working method of the team. Two community care nurses joined the team. Individual care plans were formulated with the patient and discussed during the MDT. The GP of participating patients and, if involved, community nurse were invited to the MDT. If the GP could not be present, a handover was done by phone. The patient, GP and community nurse received a copy of the individualized care plan whereas informal caregivers received information about caregiver support. The TPCT provided at least one home visit. A new colour code, ranging from green to red, was introduced to decide if the TPCT should stay involved. This was based on severity of symptoms and needs, and (im)possibilities of generalists to provide the necessary care, with green suggesting low needs and no further involvement unless requested by other professionals, and red meaning high needs and frequent involvement of the team. MDTs were continued for patients as long as the TPCT stayed involved. It is important to note that the team retained a consulting function and was never ‘in-charge’ of the patients. The team was available 24/7 for other professionals during the study period. During the study, the palliative care team received funding to compensate for the extra hours they made. GPs did not receive funding from the study itself, but there are financial constructions in place in the Netherlands where GPs can get additional payment for a so called ‘palliative care consult’ when participating in a MDT.

### Data collection

Feasibility criteria with regard to recruitment, data collection, patient burden and protocol adherence were set prior to the study (Table 2.) These criteria were assessed through quantitative data collected from the electronic medical record (EMR) and through questionnaires at baseline, 2 weeks, 1 month, 3 months and 6 months post discharge. The questionnaires included the EuroQol-5D+C [27], the Palliative outcome scale [28] the Edmonton Symptom Assessment System [29] and for caregivers the CarerQol-7D and CarerQol-VAS [30]. Furthermore, self-reported use of primary care services, which entailed hours of home care and number of (out of hour) GP visits, was reported using the questionnaires. The burden of answering the questionnaires was reported using a 10-point Likert scale, with one meaning no burden at all, and 10 meaning very high burden. Hospital-based health care utilization (number and days of hospital admissions, ER visits, palliative care team consultations) was obtained from the EMR. If applicable, place of death was obtained from the EMR. Adherence to the intervention protocol was assessed through records kept by the palliative care team. This included number and content of consultations, attendance records of the MDTs, use of colour code, if care plans were handed over, and the time at which handovers were sent. All quantitative data was collected in CASTOR Electronic Data Capture for safe and valid data collection [31].

**Table 2:** Feasibility criteria

Feasibility criteria	Criteria met
50 patients are included during 6 months	8 were included (16%)
60% of patients who meet the inclusion criteria consent to participate in the study	61% consented (8/13)
50% of patients assign an informal caregiver	62.5% assigned informal caregiver
90% completes baseline demographics and questionnaires by participants at baseline	100% complete questionnaires at baseline
80% completed primary outcome (readmission) 80% complete questionnaires by alive participants at the follow-up measure points (2 weeks and 1, 3 and 6 months post-discharge)	75% completed questionnaires, primary outcomes known for all
Burden for patients and informal caregivers to complete the questionnaires is low, median score lower than 4 on a 10 point Likert scale	Patients scored the burden of answering the questionnaires as low with a median score of 1.6 (IQR 1–3) on a 10-point Likert scale.
Patients complete all the steps of the intervention (specified in Table 1) or meet the primary endpoint (death)	Not all steps of the interventions were followed for all patients

To assess professionals’ experiences and opinions about the interventions in the PalliSupport pathway, semi-structured interviews were conducted. Participants were recruited based on their involvement in the trial components. To this end, we recruited members of the palliative care team, physicians and nurses that cared for study participants at the departments, and participants’ GPs. Interview questions were adjusted to the professionals’ role in relation to the study components. For example, department professionals were asked about their experience with identifying patients for the study, whereas GPs were asked about their experience on collaboration with the palliative care team. The interviews were audio-recorded. Furthermore, a survey was conducted amongst the training participants.

### Data analysis

Quantitative data was analysed through descriptive statistics using SPSS version 24.0 [32]. The semi-structured interviews were transcribed verbatim and were thematically analysed to explore the experiences and perspectives of professionals on the feasibility of the PalliSupport care pathway [33, 34]. Two researchers, IF and DN, independently analysed the data by reading and rereading the transcripts and coding relevant passages. Initially, an open coding scheme (inductive coding), was used. However, some codes arose from specific interview questions and thus from deductive coding. The relevant passages were structured into different themes that related to the acceptability and feasibility of the transitional care pathway. Data saturation was not sought, because the number of professionals from each setting was too limited. MAXQDA software (version 12.02) was used to extract and analyse the data [35].

## Results

### Patient baseline and outcomes

Eight patients were included. Baseline characteristics are presented in Table 3. One patient died during the index admission; a further five patients died in the following 6 months. Time between inclusion and death ranged between 2 and 79 days (mean 44.6). Three patients died at home, one in a hospice and two in the hospital. Two out of five patients died at their preferred place. Three patients had an at least one readmission.

**Table 3:** Baseline characteristics

Baseline characteristics	*n* = 8
Male (%)	5 (62.5)
Age, median (IQR)	73 (66–76)
Marital status (%)	
Married	5 (62.5)
Widowed	2 (25)
Single	1 (12.5)
Living arrangement (%)	
Independent at home	5 (62.5)
At home with help	3 (37.5)
Hospitalization in past 6 months (%)	5 (62.5)
Charlson comorbidity index, median (IQR)	6.5 (6–7.75)
Polypharmacy (*n* = 7) (%)	87.5
Prior consultation palliative care team	None

*IQR* interquartile range

### Feasibility criteria outcomes

The feasibility criterion on recruitment was not met, only 16% (*n* = 8) of anticipated patients were included. Because of this low participation number, the interpretation of the other criteria is difficult (Table 2). The number of eligible patients is unknown. A total of 23 patients, for whom the palliative care team was consulted, were excluded because these patients were already dying (7), there was a language barrier (2), no consent (5), or cognitive impairment (2), they were living outside of postal code area (5) or discharged before consent could be asked (2).

All baseline questionnaires were completed; 75% of follow-up questionnaires were completed until death or end of study. One participant did not want to continue with the questionnaires, while one patient could not answer the questionnaire because he was in the dying stage. The intended primary outcome (readmissions) and data on place of death, health care usage and palliative care team consultations were known for all patients. Protocol adherence was not met for all patients. For all discharged patients, palliative assessments, MDT meetings, colour code assignment, care plan formulation and home visits were completed. However, the MDT was not always held prior to discharge because the meetings could only be held once a week and admissions were often short. Furthermore, the GPs could not always be present during the MDT due to time restraints; however, all but one was contacted by the TPCT during the hospital admission. The medical handover was not always sent within 24 h and two patients and one caregiver did not receive their care plan or information sheet.

## Experiences and opinions of professionals

Overall, 34 professionals participated in the training modules and answered the survey. Furthermore, 14 professionals were interviewed (Table 4). Here, we present the findings on (1) training module, (2) inclusion/identification, (3) transitional palliative care team and (4) responsibility. Quotes were added to illustrate the findings.

**Table 4:** Characteristics interview participants

Organization	Gender	Age, ranged	Experience in current role, years
Division			
Respondent			
Hospital			
Pulmonary department			
1. Student nurse	F	20–29	3.0
2. Nurse	F	40–49	3.0
3. Resident	M	30–39	2.5
Gastroenterology department			
4. Student nurse	F	30–39	1.0
5. Nurse	F	30–39	2.5
6. Physician in training	M	20–29	3.0
7. Nurse team leader	F	40–49	1.0
Hospital and primary care			
Transitional palliative care team			
8. Specialist nurse	F	30–39	6.0
9. Specialist nurse	F	30–39	4.0
10. General practitioner in training	F	30–39	2.0
11. General practitioner	F	40–49	6.0
12. Community nurse	F	40–49	Unknown
Primary care			
13. General practitioner	M	50–59	20.0
14. General practitioner	F	40–49	Unknown

*M* male, *F* female

### Training module

The participants rated the training a 7.9 out of 10. The fact that the training was combined for both nurses and physicians was evaluated positively, and as contributing to collaboration on this subject. Most participants also felt the training addressed an important subject and that the training contributed to their skills. When asked for ways to improve the module, the participants suggested incorporating training on how to improve communication with patients of different cultural and religious backgrounds, allotting more training time to conversation practice and offering the training module more often to ensure maximum participation.

### Inclusion/identification

The exclusion criteria were found to be strict, which resulted in patients who could have benefitted from palliative care not being approached. Two physicians described that identification of patients for the study was not high on their priority list due to other more pressing matters during their workday. Despite the training, many physicians continued to associate palliative care with the terminal or dying stage. Nurses described being frustrated that physicians often did not agree patients were suitable for palliative care, and thus not eligible for the study, and were afraid to be turned down if they suggested otherwise.

*“But then you say, well I think that the patient does not have a year to live. Shouldn’t we be thinking about PalliSupport? By which, yes, to me it still feels a bit like I am devaluing the physician. They are still busy trying to fix the problem. To treat.”* (department nurse)

Other barriers to inclusion were respondents’ hesitation to introduce the transitional palliative care team to patients because they feared patients would react negatively. Furthermore, some respondents felt the hospital was not the best setting to hold conversations about palliative care because of short admission time, focus on cure and lack of privacy in hospital rooms. Working in shifts also made it difficult to bond with patients, which many considered a precondition for starting end of life conversations. Furthermore, nurses felt they needed approval of physicians to start end of life conversations.

*“That they (physicians) just find it scary to address the subject, I think. And as a nurse it can sometimes be very difficult to start a conversation about the end of their life when the physician says everything will be alright”.* (department nurse)

All in all, respondents felt that, despite the training, they were still late to initiate palliative care. Suggested improvements for inclusion were as follows: appointing a dedicated professional at each department that would be responsible for assessing potential participants and taking a moment each day to assess patients for potential participation.

### Transitional palliative care team

The specialist nurses and community nurse found the home visits to be very informative, yet time-intensive. The protocol stated all patients should have one home visit; however, some felt this was not necessary in all cases and should be based on the colour code assigned during the first MDT.

*“Well it’s just fun (laughter), to get on your bike and go somewhere. That’s very different from when you see someone in their (hospital) bed here. Because you really enter someone’s world. And then you walk in and you think, I see a stair lift here. Then it turns out that this man already has a stair lift. Well the stair lift is from the neighbour but he can use it himself as well. And those are the relevant things you don’t pick up on as quickly if you see someone in their (hospital) bed.”* (specialist nurse, TPCT).

Participation in the MDT by the GP was thought to improve collaboration and clarification of responsibility, but was difficult to achieve logistically. Being available after hours constituted a major time investment for the small team while the benefit was unknown and they were consulted only once by the professionals. The TPCT was also open for telephone consultation with patients and relatives. Although this was not stated in the protocol, respondents felt the phone calls increased because of the home visits.

*“But I have experienced regularly that a GP is present at the MDT. That we discuss together what would be good for a patient and that the GP is very happy with it. He (the GP) receives tailored advise from specialist. (…) So some GPs are very happy about it. And other GPs, yeah, it’s difficult, you don’t see them or hear from them.* (GP in training, TPCT )

### Responsibility

The specialist nurses of the TPCT felt a continued responsibility for patients in the study. They continued to check-up on them, even though this was not stated in the protocol. This was thought to lead to increased expectations of patients and the GPs.

*“At the same time it creates expectations, because I cannot solve everything myself and not everything belongs on my plate. So it immediately raises new questions. Like, is this my responsibility? So yes, it could have a negative side, that it creates to much expectations for a patient.”* (specialist nurse, TPCT)

One of the GPs had the expectation that the TPCT would become the main contact in the hospital for the patient. Both the GPs and the TPCT members felt the GP was primarily responsible for a patient after discharge and the home visits were perceived to be somewhat interfering with the GP’s responsibility. Therefore, discussing with the GP when and why home visits were performed was well received. The protocol however did not include how the TPCT should report back to GPs after each home visit, which was also thought to be necessary.

## Discussion

This was a mixed-method feasibility study to assess the PalliSupport care pathway prior to a stepped-wedge RCT. The protocol in its current form proved to be unfeasible because of the low recruitment rate. Additionally, protocol adherence and data collection of secondary outcomes need improvement. The training module was found to add to the professionals’ communication skills and was thought to improve patient care. Continued misunderstanding of when palliative care can be initiated, hindered the study, as well as time restraint.

### Findings and comparison to literature

Within the Medical Research Council Framework, a feasibility study is an important step in the development of a complex intervention after the developing stage [18]. Feasibility studies in palliative care are rare. While many studies are described as feasibility studies, such studies do not always include criteria to judge success or failure [21, 36]. In our study, we did formulate feasibility criteria on recruitment, data collection, patient burden and protocol adherence. The biggest setback in our study was the low recruitment rate which also influenced how we could interpret the other criteria. Low recruitment is not uncommon in palliative care studies [37]. A review on cancer studies by Grand et al. determined that obstacles for accrual can be found in three different categories: clinician, patient and system [38]. In our study, clinicians seem to have formed the biggest barrier to recruitment because the number of times the TPCT was called was considerably lower than anticipated and if they were called, many patients were already dying. Unfortunately, because of strict privacy laws in this particular hospital, researchers could not access patients’ records to assess potential participants themselves. Clinicians are often hesitant to approach patients and informal caregivers with a request for study participation in such a vulnerable time in the patients’ lives, and some doubt it is even ethical to do so [39, 40]. However, patients and caregivers in our study did not report their participation to be a heavy burden. Although inclusion criteria were set for participation, clinicians’ own assessment of the need for palliative care seemed to overrule these set criteria. Physicians, in particular, still associate palliative care with the terminal or dying stage and in our study therefore did not suggest patients for participation [41]. Furthermore, while nurses seem to identify patients earlier in their trajectories, they can be hesitant to disclose these findings to physicians [42]. We therefore have to conclude that solely relying on department clinicians to enrol patients for participation is not feasible.

Baseline data collection and data on the RCT’s primary outcome (re-hospitalization) were achieved for all patients. Our secondary outcomes depended on the completion of follow-up questionnaires. However, these were not always completed. This is not surprising. When patients are nearing death, answering questionnaires becomes more difficult. Assessing quality of life through questionnaires with relatives, such as the Quality of Dying and Death Questionnaire, could overcome this data gap [43]. However, when choosing primary outcomes measures in palliative care, more practical measures, such as hospitalization or place of death, increase the success rate of follow-up.

Protocol adherence was based on previous formulated steps that had to be met for each patient. The different steps of the intervention were not always followed according to our intended sequence and in the intended manner, although this conclusion is based on limited data. In some cases, this was limited to the MDT being post-discharge. In another case, the GP was not contacted, and in yet other cases, the care plan was not provided to patients which both could potentially influence the effectiveness outcomes. Care pathways are by definition complex interventions and need to be adjusted to the structures already in place [44]. Complete adherence to all parts of the protocol might therefore not be achievable. When evaluating the effectiveness of care pathways, a process evaluation can contribute to the understanding of what is implemented and to what extent this influences effectiveness outcomes [44–46].

Our qualitative data yielded insight into another aspect that could hinder our RCT: difficulty in transitional collaboration and division of roles. Lack of collaboration between specialists and generalists in palliative care has been a frequent occurrence [47, 48]. In the Netherlands, the GP is the gatekeeper and most often the primary physician during the last phase of life. This responsibility is temporarily transferred to medical specialists when patients enter the hospital. The transitional palliative care team aimed to act as a bridge between the two settings and to provide transmural consultation. However, both the transitional palliative care team and GPs feared that, because of the home visits, the team would take over the care for the patient at home. This could lead to an unnecessary power struggle between the two which could be an important barrier to the success of the intervention.

### Implications for the effectiveness trial

Based on the findings of this feasibility study, the protocol for the ongoing stepped-wedge RCT has been adjusted. First, to improve inclusion rates during the RCT, instead of waiting for clinicians, researchers are now actively screening the admission records for potential participants and asking the Surprise Question within the daily rounds [49]. Second, an e-learning on timely identification and starting conversation about the end of life has been added to the training program. Third, a questionnaire for relatives on quality of dying and death has been included.

Fourth, to improve collaboration between primary and secondary care, the set-up of the transitional care pathway has been adjusted so that GPs, medical specialists, nurses and the palliative care team are now all involved from the start. We have also started identifying existing regional structures for palliative care building upon these structures. In addition, prior to the intervention phase of the study, meetings are being held between the study coordinators, palliative care teams and primary care organizations to make collaboration agreements and to enhance familiarity with the project.

Fifth, to lower the burden for the TPCTs, the 24/7 availability has been removed from the trial. Finally, we have adopted a rigorous mixed-method process evaluation according to the MRC framework alongside the stepped wedge RCT [18].

### Strengths and limitations

This feasibility study was designed to identify potential shortcoming in a study protocol for a stepped-wedge RCT in correspondence with the tutorial by Thabane et al. [21]. This led to valuable adjustments in the research protocol. However, the design of the feasibility study itself could also have been improved. Including experiences and perspectives of patients on the project could have given a different perspective. Furthermore, we could not collect data on all eligible patients but only on those for whom the TPCT was called. Therefore, we do not known the actual number of potential participants. Additionally, this was a feasibility study with one study site. We do not expect major differences in feasibility in other sites. However, we cannot be certain that all results can be generalized to other geographical settings. Regional implementation barriers and facilitators will be investigated as part of a comprehensive process evaluation of the stepped wedge RCT.

## Conclusion

The PalliSupport care pathway protocol outlined in this paper needs to be adjusted to improve recruitment, protocol adherence and data collection at follow-up. When developing a complex intervention in a palliative care setting, such as a care pathway, it is advised to perform a thorough feasibility study before embarking on larger trials. Special attention should be given within the study protocol to recruitment and how to involve clinicians in this process. Data collection can be challenging during follow-up because of the fragile condition of the participants and outcome measures should be chosen deliberately. Process evaluations should be a part of your trial to determine which aspects of an intervention work within the existing structures and how implementation will affect the effectiveness outcomes.

## Data Availability

The datasets generated and analysed during the current study are not publicly available due to privacy of patients and interview participants, but are available from the corresponding author on reasonable request.
